# Assessment of rabbit spermatozoa characteristics after amygdalin and apricot seeds exposure *in vivo*

**DOI:** 10.1016/j.toxrep.2018.05.015

**Published:** 2018-05-31

**Authors:** Eduard Kolesar, Eva Tvrda, Marek Halenar, Monika Schneidgenova, Lubica Chrastinova, Lubomir Ondruska, Rastislav Jurcik, Anton Kovacik, Eva Kovacikova, Peter Massanyi, Adriana Kolesarova

**Affiliations:** a Faculty of Biotechnology and Food Sciences, Slovak University of Agriculture in Nitra, Tr. A. Hlinku 2, 949 76 Nitra, Slovak Republic; b Animal Production Research Centre Nitra, National Agricultural and Food Center, Hlohovecká 2, 951 41 Lužianky, Slovak Republic; c Research Centre AgroBioTech, Slovak University of Agriculture in Nitra, Tr. A. Hlinku 2, 949 76 Nitra, Slovak Republic

**Keywords:** Amygdalin, Apricot seeds, Spermatozoa, Rabbit

## Abstract

This study evaluates rabbit spermatozoa motility parameters after *in vivo* administration of amygdalin and apricot seeds during a 28-day period. Apricot seeds are potentially useful in human nutrition and amygdalin is the major cyanogenic glycoside present therein. The rabbits were randomly divided into the five groups (Ctrl-Control, P1, P2, P3, P4) with 4 males in each group. Control group received no amygdalin/apricot seeds while the experimental groups P1 and P2 received a daily intramuscular injection of amygdalin at a dose 0.6 and 3.0 mg/kg b.w. respectively during 28 days. P3 and P4 received a daily dose 60 and 300 mg/kg b.w. of crushed apricot seeds mixed with feed during 28 days, respectively. CASA system was used to evaluate for motility, progressive motility, curvilinear velocity, amplitude of lateral head displacement and beat cross frequency. Intramuscular application of amygdalin resulted in a significant time- and dose-dependent decrease of spermatozoa motility as well as progressive motility. On the other hand, oral consumption of apricot seeds had no significant effect either on the rabbit spermatozoa motility or progressive motility over the entire course of the study. The analysis of the other motion characteristics revealed a similar trend depicting a continuous, time- and dose-dependent decrease of all parameters following intramuscular AMG administration, with significant differences particularly for the dose 3.0 mg AMG/kg b.w. On the other hand, oral administration of apricot seeds had no significant impact on spermatozoa motility parameters. The present study suggests that short-term intramuscular application of amygdalin decreased rabbit spermatozoa motility *in vivo*. Whereas, consumption of apricot seeds did not induce any change in rabbit spermatozoa *in vivo*. Our findings suggest dose-dependent negative effect of pure amygdalin, but not apricot seeds on the rabbit spermatozoa parameters.

## Highlights

• Rabbit spermatozoa parameters after amygdalin and apricot seeds exposure.
• Evaluation of spermatozoa motility by the CASA system.
• Decrease of spermatozoa motility after intramuscular AMG application.
• Oral consumption of apricot seeds had no effect on the spermatozoa motility.
• Our data suggest the potential impact of AMG and apricot seeds on male reproduction.

## 1 Introduction

More than 50% of cancer patients in Europe use complementary/alternative medicine (CAM) instead of or combined with conventional therapy [1]. Amygdalin (AMG) has been one of the most popular “alternative cancer cures” in many European and South American countries [2]. AMG (D-mandelonitrile-β-d-gentiobioside) is a cyanogenic glycoside found in variety of plant species, mainly in the seeds of apricots and bitter almonds [3]. Seeds contain AMG depending on the variety: approximately 20–80 μmol/g AMG may be found in apricot seeds, and its concentration is very high (5.5 g/100 g) in bitter apricot cultivars while it is not detected in the sweet ones [4]. This natural substance is composed of two molecules of glucose, one benzaldehyde, and one hydrocyanic acid [5]. AMG itself is non-toxic, but it is decomposed by several enzymes into hydrogen cyanide (HCN), which is a poisonous substance [6], causing potential toxicity issues for animals including humans [7]. Diverse studies have reported on the beneficial properties of AMG and its effective usage in the prevention or treatment of various diseases including cancers, migraine, chronic inflammation, fever and pain [8], [9]. However, AMG as a therapeutic agent has not yet received FDA (Food and Drug Administration) approval for its use in the United States owing to insufficient clinical verification of its therapeutic efficacy; hence the anticancer effect of amygdalin remains controversial [2] The possible impact of different naturally cyanide-containing substances on the male reproductive system, focused on spermatozoa motility and morphological abnormalities in bull spermatozoa, was observed previously by Tanyildizi and Bozkurt [10]. The treatment of bull semen samples with amygdalin significantly (P < 0.01) inhibited the hyaluronidase activity of spermatozoa *in vitro*.

The present study was designed to reveal whether short-term intramuscular application of AMG and oral application of apricot seeds causes changes in rabbit spermatozoa *in vivo*.

## 2 Material and methods

### 2.1 Chemicals

AMG from apricot kernels (≥99% purity) was purchased from Sigma-Aldrich (St. Louis, MO, USA). AMG was freshly dissolved in sterile saline and 0.5 ml were applied intramuscularly (IM) to *musculus biceps femoris* on adaily basis. Bitter apricot seeds were provided by Trasco (Žiar n. Hronom, Slovakia). Thin Layer Chromatography (TLC) was performed for the analysis of AMG content in bitter apricot seeds used in our experiment. Chemical composition of the apricot seeds is shown in Table 1.

**Table 1:** Chemical composition of apricot seeds.

Organic content	%	Mineral content	mg/kg	Celulose components	%	Fatty acids	%
Dry matter	95.9	**Ca**	1774	**ADF**	38.8	**Palmitic acid**	4.6
Amygdalin	5.2	**P**	4700	**NDF**	45.1	**Palmitoleic acid**	0.8
N-compounds	22.8	**Mg**	2050	**Lignin**	11.7	**Steraic acid**	1.2
Fat	39.7	**Na**	642	**Celulose**	27.1	**Oleic acid**	64.5
Fiber	28.5	**K**	5925	**Hemicelulose**	6.3	**Linoleic acid**	27.1
Ash	2.5	**Cu**	14.7			**Arachidic acid**	0.1
NFE	2.4	**Fe**	24.8			**cis-11-eicosenoic acid**	0.1
OM	93.5	**Mn**	5.9			**PUFA**	27.1
Starch	2.3	**Zn**	59.7			**MUFA**	65.3
Sugar	6.3					**SFA**	5.9

NFE-nitrogen-free extract, OM- organic matter, ADF-acid detergent fiber, NDF-neutral detergent fiber, PUFA-polyunsaturated fatty acids, MUFA-monounsaturated fatty acids, SFA-saturated fatty acids.

### 2.2 Animals

Meat line P91 Californian rabbit males (n = 20) from the experimental farm of the Animal Production Research Centre Nitra (Slovak Republic) were used in the experiments. The rabbits were 150 days old, weighing 4.00 ± 0.5 kg, and were housed in individual flat-deck wire cages under a constant photoperiod of 12 h of daylight, temperature 20–24 °C and humidity 55% ± 10%. The rabbits were fed a standard commercially available feed (Table 2) based on a pelleted concentrate. Animals had free access to feed and water during the study period and no toxic or side effects or death was observed throughout the study. The animals were randomly divided into the five groups (Ctrl-Control, P1, P2, P3, P4 – experimental groups), leading to 4 male rabbits in each group. The control group received no amygdalin/apricot seeds while the experimental groups P1 and P2 received a daily intramuscular injection of amygdalin at a dose 0.6 and 3.0 mg/kg b.w. respectively during 28 days. Experimental groups P3 and P4 received a daily dose 60 and 300 mg/kg b.w. of crushed apricot seeds mixed with feed during 28 days, respectively. The intramuscular and oral doses of amygdalin were calculated to not exceed the Acute medium lethal oral doses (LD50) values for cyanide, which range from 2.13 to 6 mg/kg b.w., considering that 1 g of amygdalin release 59 mg HCN [11], [12]. The intramuscular doses (0.6 and 3.0 mg/kg b.w.) of amygdalin release 0.035 and 0.177 mg/kg HCN, respectively. Based on the amygdalin content in apricot seeds, the experimental groups P3 and P4 received 3.12 and 15.6 mg/kg b.w. of amygdalin, corresponding to 0.18 and 0.92 mg/kg HCN, respectively [13]. The body weight of each experimental animal was recorded weekly during the whole study. Institutional and national guidelines for the care and use of animals were followed appropriately, and all experimental procedures were approved by the State Veterinary and Food Institute of Slovak Republic, no. 3398/11–221/3 and Ethic Committee.

**Table 2:** Nutritional composition of the experimental diet (%).

Component	%
Dehydrated Lucerne meal	36
Extracted sunflower meal	5.5
Extracted rape seed meal	5.5
Barley grains	8.0
Oats	13.0
DDGS-dried distillers grains with solubles	5.0
Malt sprouds	15.0
Wheat bran	9.0
Sodium chloride	0.3
Minerals and Vitamins^a^	1.7
Limestone	1.0

a Provided per kg diet: vit. A 12,000 IU; vit.D_2_ 2500 IU; vit. E 20 mg; vit.B_1_ 1.5 mg;vit. B_2_ 7.5 mg;vit. B_6_ 4.5 mg; vit. B _12_ 30 μg; vit.K 3 mg; nicotic acid 45 mg; folic acid 0.8 mg; biotin 0.08 mg ; Choline chloride 450 mg; Premix minerals (per kg diet) cca 9.25 g; P 6.2 g; Na 1.6 g; Mg 1.0 g; K 10.8 g; Fe 327.5 mg; Mn 80 mg; Zn 0.7 mg.

### 2.3 Semen samples

Semen samples from males of control and experimental groups were collected weekly on the same day (early in the morning) using an artificial vagina [14]. Immediately after collection each sample was diluted in physiological saline solution (PS) (sodium chloride 0.9% w/v, Bieffe Medical, Italia) using a dilution ratio of 1:5.

Spermatozoa motility was examined with the help of the CASA system using the SpermVision™ program (Minitube, Tiefenbach, Germany) and Olympus BX 51 phase contrast microscope (Olympus, Tokyo, Japan). The samples were placed into the Makler counting chamber (depth 10 μm, 37 °C; Sefi Medical Instruments, Haifa, Israel) and immediately assessed [15]. Thousand cells were evaluated in each sample for the following characteristics: motility (percentage of cells moving faster than 5 μm/s; %), progressive motility (percentage of cells moving faster than 20 μm/s; %), curvilinear velocity (VCL, μm/s), amplitude of lateral head displacement (ALH, μm) and beat cross frequency (BCF, Hz) [15], [16], [17], [18].

### 2.4 Statistical analysis

Statistical analysis was carried out using the GraphPad Prism program (version 3.02 for Windows; GraphPad Software, La Jolla California USA, www.graphpad.com). Descriptive statistical characteristics (mean, standard error) were evaluated at first. One-way ANOVA was used for specific statistical evaluations. Dunnett test was used as a follow-up test to ANOVA, based on a comparison of every mean to a control mean, and computing a confidence interval for the difference between the two means. The level of significance was set at P < 0.001, P < 0.01, and P < 0.05.

## 3 Results

**Fig. 1:**
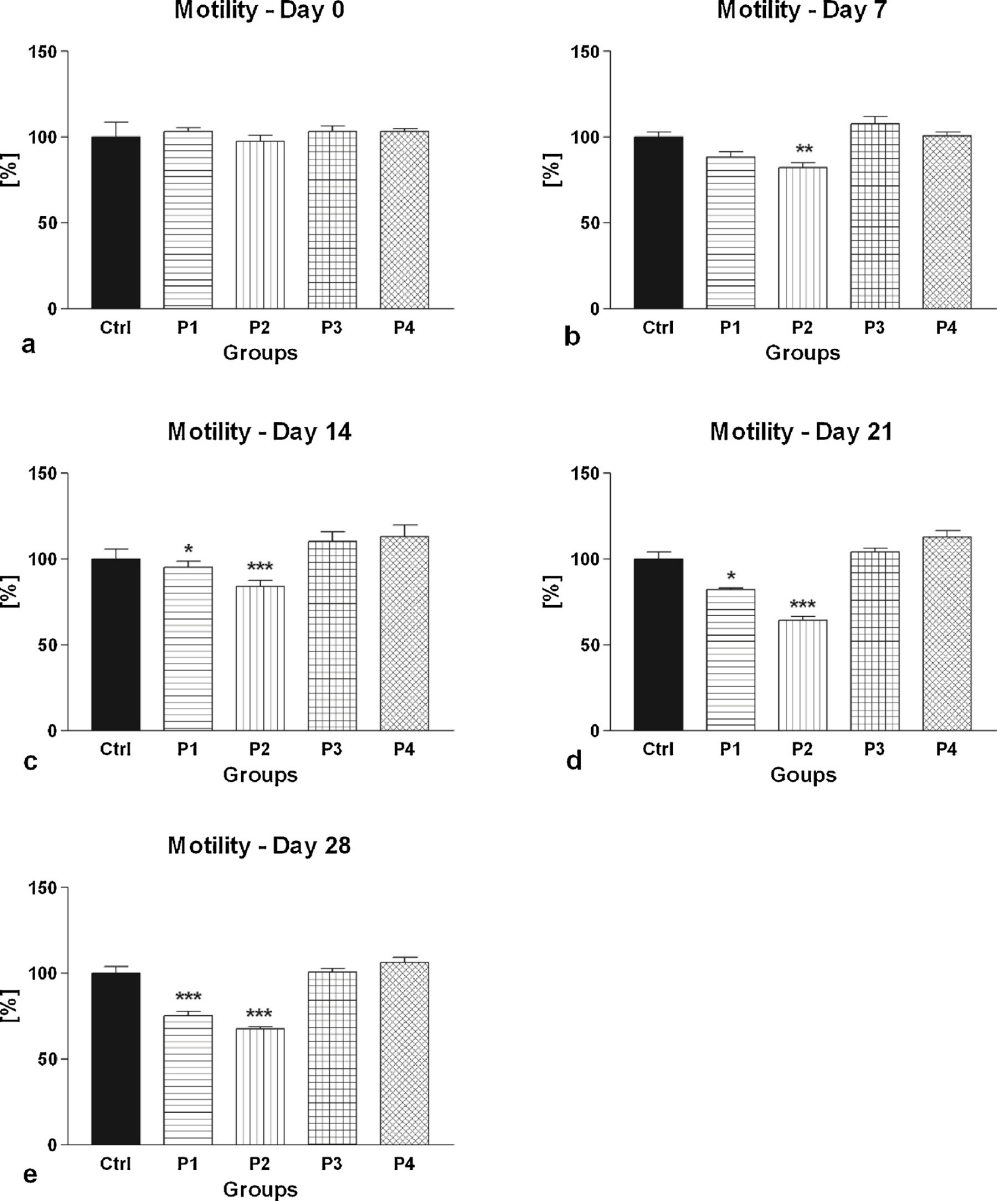
(a–e) The effect of intramuscular (groups P1 and P2) and oral (groups P3 and P4) amygdalin administration on rabbit spermatozoa motility after 0 (a), 7 (b), 14 (c), 21 (d) and 28 (e) days of treatment. Ctrl – Control group; P1 – 0.6 mg AMG/kg b.w. intramuscular administration; P2 – 3.0 mg AMG/b.w. intramuscular administration; P3 – 60 mg apricot seeds/kg b.w. oral administration; P4 – 300 mg apricot seeds/kg b.w. oral administration. *P < 0.05; **P < 0.01; ***P < 0.001. Thousand cells were evaluated in each sample for the motility.

**Fig. 2:**
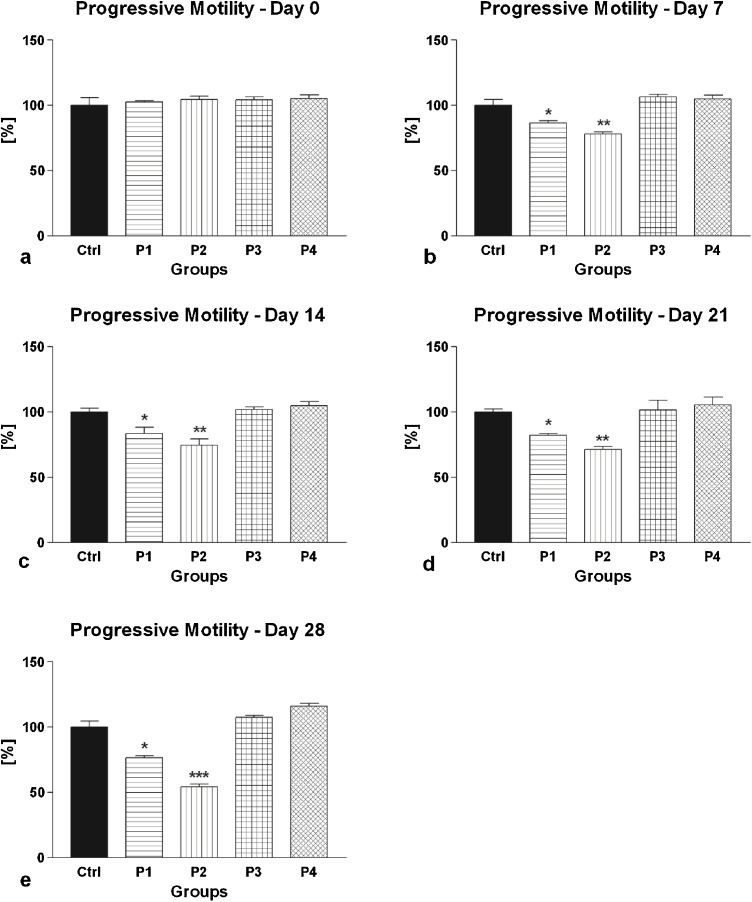
(a–e) The effect of intramuscular (groups P1 and P2) and oral (groups P3 and P4) amygdalin administration on rabbit spermatozoa progressive motility after 0 (a), 7 (b), 14 (c), 21 (d) and 28 (e) days of treatment. Ctrl – Control group; P1 – 0.6 mg AMG/kg b.w. intramuscular administration; P2 – 3.0 mg AMG/b.w. intramuscular administration; P3 – 60 mg apricot seeds/kg b.w. oral administration; P4 – 300 mg apricot seeds/kg b.w. oral administration. *P < 0.05; **P < 0.01; ***P < 0.001. Thousand cells were evaluated in each sample for the progressive motility.

**Fig. 3:**
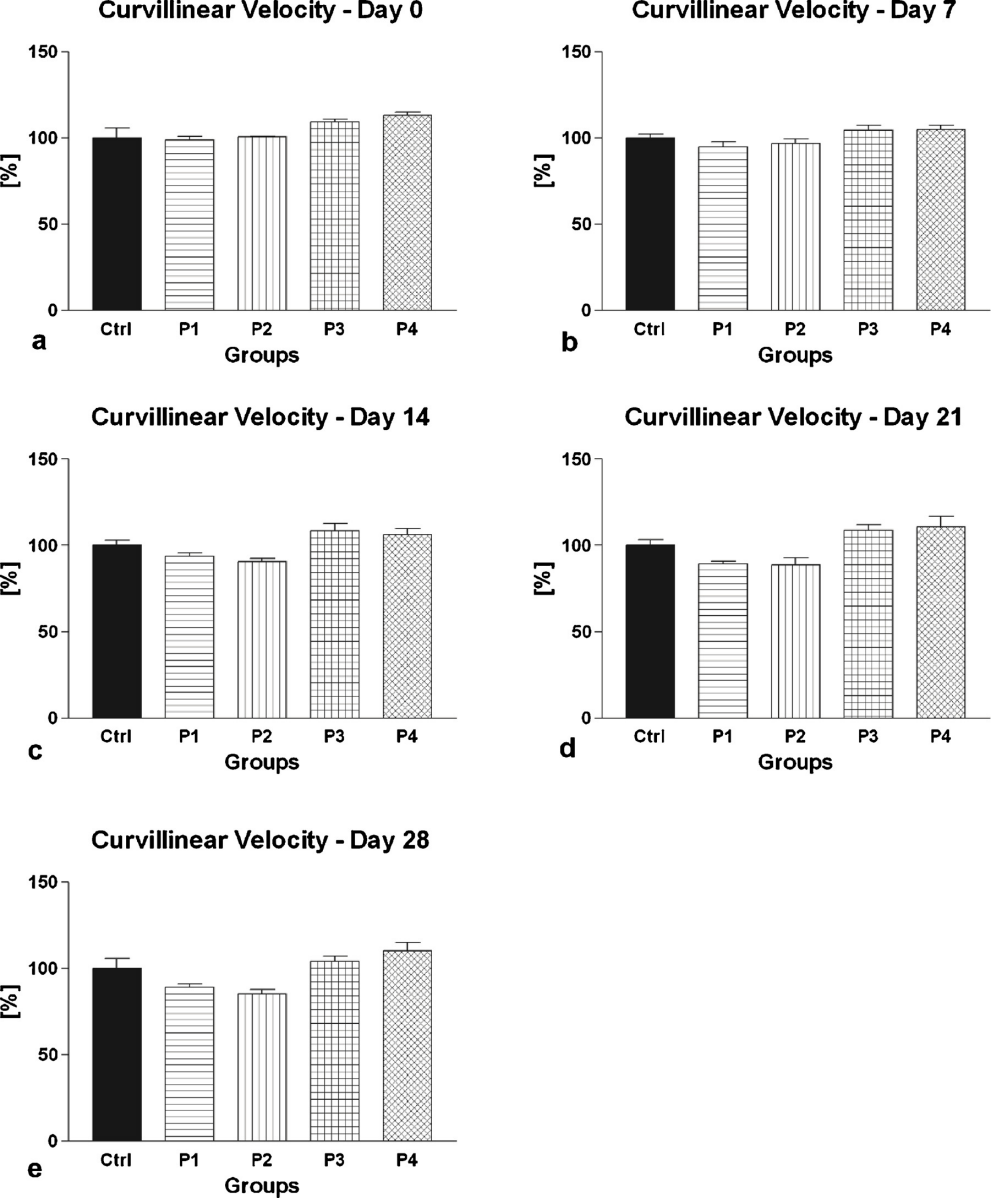
(a–e) The effect of intramuscular (groups P1 and P2) and oral (groups P3 and P4) amygdalin administration on rabbit spermatozoa curvilinear velocity after 0 (a), 7 (b), 14 (c), 21 (d) and 28 (e) days of treatment. Ctrl – Control group; P1 – 0.6 mg AMG/kg b.w. intramuscular administration; P2 – 3.0 mg AMG/b.w. intramuscular administration; P3 – 60 mg apricot seeds/kg b.w. oral administration; P4 – 300 mg apricot seeds/kg b.w. oral administration. *P < 0.05; **P < 0.01; ***P < 0.001. Thousand cells were evaluated in each sample for the curvilinear velocity.

**Fig. 4:**
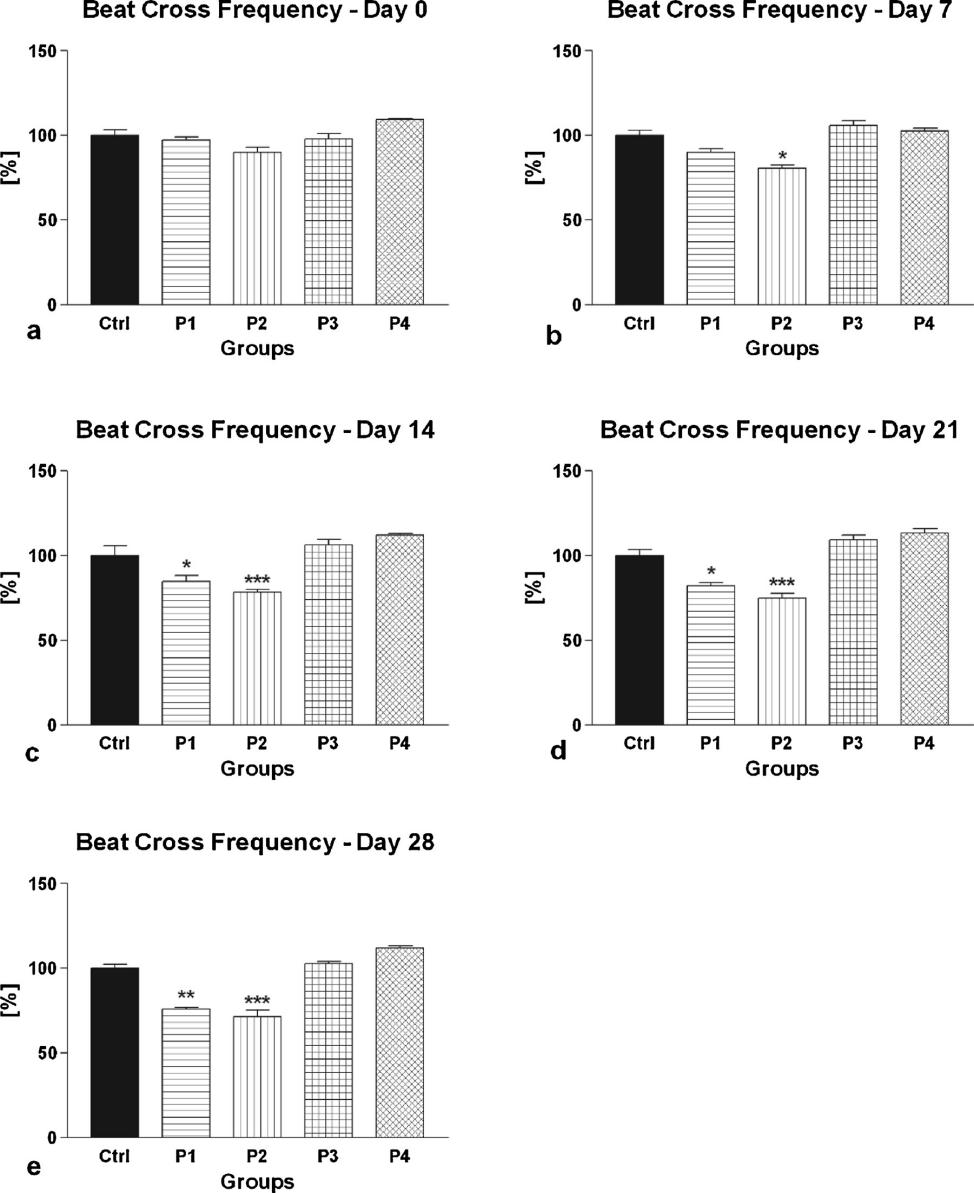
(a–e) The effect of intramuscular (groups P1 and P2) and oral (groups P3 and P4) amygdalin administration on rabbit spermatozoa beat cross frequency after 0 (a), 7 (b), 14 (c), 21 (d) and 28 (e) days of treatment. Ctrl – Control group; P1 – 0.6 mg AMG/kg b.w. intramuscular administration; P2 – 3.0 mg AMG/b.w. intramuscular administration; P3 – 60 mg apricot seeds/kg b.w. oral administration; P4 – 300 mg apricot seeds/kg b.w. oral administration. *P < 0.05; **P < 0.01; ***P < 0.001. Thousand cells were evaluated in each sample for the beat cross frequency.

**Fig. 5:**
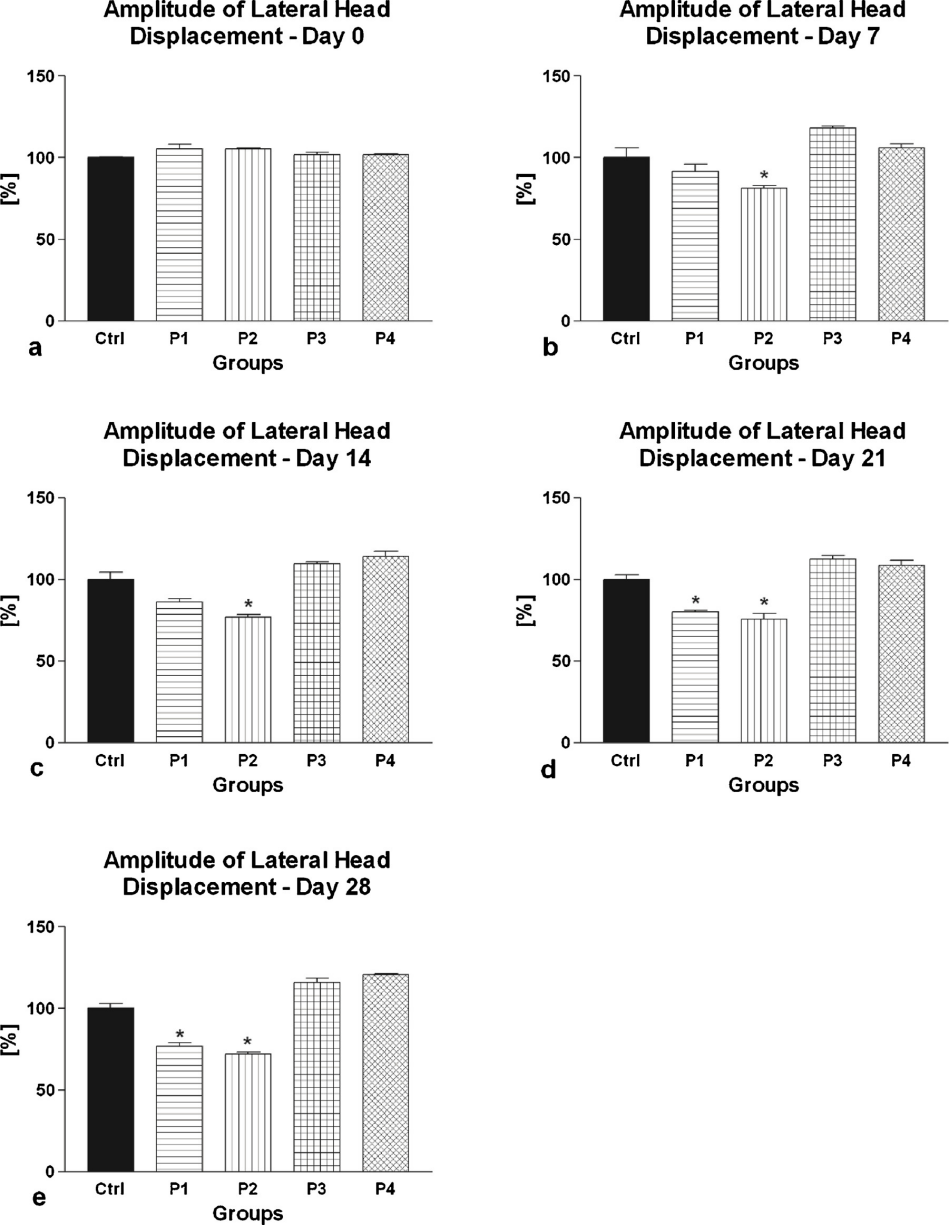
(a–e). The effect of intramuscular (groups P1 and P2) and oral (groups P3 and P4) amygdalin administration on rabbit spermatozoa amplitude of lateral movement after 0 (a), 7 (b), 14 (c), 21 (d) and 28 (e) days of treatment. Ctrl – Control group; P1 – 0.6 mg AMG/kg b.w. intramuscular administration; P2 – 3.0 mg AMG/b.w. intramuscular administration; P3 – 60 mg apricot seeds/kg b.w. oral administration; P4 – 300 mg apricot seeds/kg b.w. oral administration. *P < 0.05; **P < 0.01; ***P < 0.001. Thousand cells were evaluated in each sample for the amplitude of lateral movement.

The primary as well as secondary motility characteristics of spermatozoa collected from the control as well as experimental groups are shown in Fig. 1, Fig. 2, Fig. 3, Fig. 4, Fig. 5.

The CASA motility analysis showed that intramuscular AMG administration resulted in a significant time- and dose-dependent decrease of spermatozoa motility (Fig. 1) as well as progressive motility (Fig. 2) in comparison to the control group. On the other hand, oral consumption of apricot seeds had no significant effect neither on the rabbit spermatozoa motility or progressive motility over the entire course of the *in vivo* experiment. At the end of the study, the lowest motility and progressive motility were recorded in the experimental group P2, subjected to intramuscular administration of 3.0 mg/kg AMG, followed by the experimental group P1, which received 0.6 mg AMG/kg intramuscularly. In both cases, the motility as well as progressive motility were significantly decreased when compared to the control group. Meanwhile, the experimental groups supplemented with apricot seeds exhibited the highest motility and progressive motility, however without significant differences when compared to the control.

The analysis of the secondary (additional) motility characteristics revealed a similar trend depicting a continuous, time- and dose-dependent decrease of VCL, ALH or BCF following intramuscular AMG administration, with significant differences particularly in the case of 3.0 mg/kg AMG (Fig. 3, Fig. 4, Fig. 5). Inversely, oral administration of apricot seeds had no significant impact on all parameters in comparison to the control group.

## 4 Discussion

Previous studies describe the effect of AMG on reproductive functions in animals [19], [20], [21], [22], [13], [23], [10]. In our *in vivo* study on rabbit model the effects of intramuscular application of AMG and oral consumption of apricot seeds were evaluated. Firstly, the intramuscular AMG administration resulted in a significant time- and dose-dependent decrease of spermatozoa motility as well as progressive motility. The analysis of the secondary motion characteristics revealed a similar trend depicting a continuous, time- and dose-dependent decrease of all parameters following intramuscular AMG administration. Similarly, as shown in previous study, the hyaluronidase activity was inhibited significantly by low concentrations of AMG (P < 0.01) (0.4–2 μM) [10]. Additionally, linamarin a cyanogenic glycoside found in a variety of plant, including cassava and lima beans, caused significant decrease in bull spermatozoa motility. It has been reported that bull spermatozoa heads contain a beta-type DNA polymerase enzyme [24]. Previous study describes that the activities of DNA polymerase alpha, beta and gamma were significantly lower in infertile men than in normal controls [25]. Additionally, Mizushina et al. [26] noted that amygdalin glycoside dose-dependently inhibited the activity of rat DNA polymerase beta. The previous findings suggest that all spermatozoa lost their motility and were immobile at 10 min in a dose- dependent manner [10]. We confirm previous findings [10] that the decrease in spermatozoa motility may be inhibited by AMG treatment. On the other hand, the *in vitro* study of Halenar et al. [22] suggests that short-term AMG supplementation has no negative effects on the rabbit spermatozoa survival *in vitro*. It may be suggested that glucose may be the first molecule to be released from AMG and to subsequently stimulate the mitochondrial metabolism followed by the motion activity of rabbit spermatozoa [22]. A recent study revealed that AMG may have a dose-dependent activity on the testicular tissue, displaying an interesting dichotomy: low doses may improve the oxidative balance, yet high doses may compromise this delicate milieu [19].

Second, oral consumption of apricot seeds had no significant effect neither on the rabbit spermatozoa motility and/or progressive motility over the entire course of the *in vivo* experiment. The analysis of the secondary motility characteristics revealed that oral administration of apricot seeds had no significant impact on secondary parameters. A previous study describes that the fertilizing ability of bull spermatozoa can be inhibited by the excessive consumption by bulls of diets containing cyanogenic plants and cotton seed [10]. On the other hand, apricot seed is an important source of dietary protein along with a significant amount of oil and fibers and exhibited higher antioxidative activity then flesh of the fruit [27]. Based on previous studies [27], [28] it may be assumed that apricot seeds provide significant protective activity. Similarly, doses of apricot seed used in our study do not exhibit harmful effect on rabbit spermatozoa parameters. On the other hand, pure form of amygdalin may represent potential risk for male reproductive system depending on the used doses. We suppose that complex of various compounds present in apricot seeds may be the cause of different action of pure amygdalin form and apricot seeds administration.

In according with EFSA [11] AMG as the major cyanogenic glycoside present in apricot seeds is degraded to cyanide by chewing or grinding. Cyanide is of high acute toxicity in humans. On the other hand, animal data did not provide a suitable basis for acute human health hazard assessment. The CONTAM Panel of EFSA [11] concluded that the lethal dose is reported to be 0.5–3.5 mg/kg body weight (b.w.). An acute reference dose (ARfD) of 20 μg/kg b.w. was derived from an exposure of 0.105 mg/kg bw associated with a non-toxic blood cyanide level of 20 μM, and applying an uncertainty factor of 1.5 to account for toxicokinetic and of 3.16 to account for toxicodynamic inter-individual differences.

In our study short-term consumption of apricot seeds at the doses 60 and 300 mg/kg b.w. did not confirm toxic effect of apricot seeds on rabbit spermatozoa *in vivo*.

## 5 Conclusion

The present study suggests that short-term AMG supplementation decreased rabbit spermatozoa motility *in vivo*. On the other hand, consumption of apricot seeds did not induce changes in rabbit spermatozoa *in vivo*. Our findings suggest dose-dependent negative effect of pure amygdalin, but not apricot seeds on the rabbit spermatozoa parameters. Our data may provide more specific evidence to unravel the behavior of AMG in male reproduction.

## Declaration of interest

The authors have reported that no competing interests exist.
